# The effect of hospital volume on length of stay, re-admissions, and complications of total hip arthroplasty: A population-based register analysis of 72 hospitals and 30,266 replacements

**DOI:** 10.3109/17453674.2010.533930

**Published:** 2011-02-10

**Authors:** Keijo T Mäkelä, Unto Häkkinen, Mikko Peltola, Miika Linna, Heikki Kröger, Ville Remes

**Affiliations:** ^1^Department of Orthopaedics and Traumatology, Turku University Central Hospital; ^2^National Institute for Health and Welfare; ^3^Department of Orthopaedics and Traumatology, Kuopio University Hospital; ^4^Department of Orthopaedics and Traumatology, Peijas Hospital, Helsinki University Central Hospital, Finland

## Abstract

**Background and purpose:**

Hospital volume has been suggested to be one of the best indicators of adverse orthopedic events in patients undergoing THR surgery. We therefore evaluated the effect of hospital volume on the length of stay, re-admissions, and complications of THR at the population level in Finland.

**Methods:**

30,266 THRs performed for primary osteoarthritis were identified from the Hospital Discharge Register. Hospitals were classified into 4 groups according to the number of THRs performed on an annual basis over the whole study period: 1–50 (group 1), 51–150 (group 2), 151–300 (group 3), and > 300 (group 4).

**Results:**

In 2005, the length of the period of surgical treatment was 5.5 days in group 4 and 6.8 days in group 1 (the reference group). During the whole study period (1998–2005), the length of surgical treatment period was shorter in group 4 than in group 1 (p < 0.001). The odds ratio for dislocations (0.7, 95% CI: 0.6–0.9) was lower in group 3 than in group 1.

**Interpretation:**

Hip replacements performed in high-volume hospitals reduce costs by shortening the length of stay, and they may reduce the dislocation rate.

The association between hospital volume and results of total hip replacement (THR) has been investigated in several studies (Lavernia and Guzman 1995, Battaglia et al. 2006, Doro et al. 2006, Shervin et al. 2007). The surgeon volume and the hospital volume have been suggested to be the best indicators of adverse orthopedic events in patients undergoing THR surgery (Solomon et al. 2002). A systematic review of the literature found an association between high hospital volumes and low numbers of hip dislocations (Shervin et al. 2007). Lower provider volumes have been related to longer hospital stay after THR surgery (Lavernia and Guzman 1995, Doro et al. 2006, Judge et al. 2006).

We evaluated the effects of hospital volume on the length of stay, the number of re-admissions, and the number of complications of THRs in the whole population of Finland.

## Patients and methods

All public and private hospitals in Finland are obliged to report any surgical procedures requiring an overnight stay to the National Institute for Health and Welfare. The Hospital Discharge Register, which is maintained by the National Institute for Health and Welfare, was the main database we used. All other data were obtained from the following sources: the Benchmarking database compiled for the Hospital Benchmarking project for productivity in specialized care (Linna and Häkkinen 2008), the Social Insurance Institution database, and the Finnish Arthroplasty Register. The characteristics of the data derived from these registries are dealt with later. Every Finnish resident has a unique personal identification number, which can be used to combine data from different registers.

The effect of hospital volume on the length of the surgical treatment period (referred to later as length of stay (LOS)), the length of uninterrupted institutional care (LUIC), and the rates of unscheduled re-admissions, reoperations, dislocations, and infections were evaluated. The surgical treatment period was defined as the period during which the THR was performed in the hospital, as shown by the Hospital Discharge Register. A surgical treatment period ended in discharge, in transfer to another facility, or in death of the patient. Uninterrupted institutional care was defined as the combination of the surgical treatment period and the immediate period of rehabilitation. Uninterrupted institutional care ended in either death or discharge of the patient, which also included the patient being transferred to another facility such as an old people's home or another long-term care or social welfare institution. The maximum length of institutional care was limited to 60 days as a cutoff point in the calculations. The study period was from 1998 through 2005. Reoperations, closed and open reductions of dislocated hip prostheses, and infections of the THR were followed to the end of 2008.

### Hospital grouping

Hospitals were classified into 4 groups according to the number of THRs (NOMESCO codes NFB30–NFB99) performed annually during the whole study period: 1–50 (low-volume hospitals, group 1), 51–150 (medium-volume hospitals, group 2), 151–300 (high-volume hospitals, group 3), and > 300 (very-high-volume hospitals, group 4) (Table 1). The low-volume hospitals were used as a reference group.

**Table 1.** Number of patients and annual number of hospitals in different hospital volume groups, with annual number of private hospitals in parentheses

Group	No. of patients 1998–2005	Annual no. of hospitals							
		1998	1999	2000	2001	2002	2003	2004	2005
1	2,759	15 (7)	18 (8)	23 (8)	25 (9)	23 (10)	19 (11)	19 (10)	18 (9)
2	11,591	29 (2)	32 (2)	26 (2)	26 (3)	28 (2)	22 (2)	21 (2)	23 (4)
3	8,134	2 (0)	9 (0)	9 (0)	8 (0)	10 (1)	9 (0)	10 (0)	8 (0)
4	7,782	1 (1)	2 (1)	4 (1)	5 (1)	3 (1)	7 (2)	6 (2)	9 (2)
Total	30,266	57 (10)	61 (11)	62 (11)	64 (13)	64 (14)	57 (15)	56 (14)	58 (15)

See Material and Methods for explanation of groups

There were 72 hospitals involved. Hospitals that changed hospital volume group during the study period were also included. 39 hospitals did not change volume group (9,926 hips) and 33 hospitals changed volume group once (20,340 hips). None of the hospitals changed volume group more than once.

### Inclusion criteria

The study population was formed by selecting patients from the Hospital Discharge Register according to the International Classification of Diseases (ICD-10), and the following were used as selection criteria: M16.0/M16.1 for primary osteoarthritis (OA) of the hip or M16.2/M16.3 for developmental dysplasia of the hip (DDH), associated with a code for primary THA performed over the 1998–2005 period. Patients with a diagnosis of developmental dysplasia of the hip (DDH) (ICD-10: M16.2/M16.3) were included in the study because there is variation in coding of mild DDH and primary OA. Codes for THR included NFB30 for cementless THR, NFB40 for hybrid THR, NFB50 for cemented THR, NFB60 for demanding THR, and NFB99 for other THR procedures such as hip resurfacing. Patients with congenital hip dislocation are classified under a different diagnosis code (Q65.0-Q65.9) and were not included in the study. The accuracy of diagnosis for primary OA was double-checked against the relevant data in the Finnish Arthroplasty Register. Total hip replacements—not patients—were evaluated when considering the length of surgical treatment period, the length of institutional care, and unscheduled re-admissions. Thus, it was possible that 2 THRs were evaluated from the same patient. The total number of all the THRs included was 30,266. However, when considering reoperations, dislocations, and infections, only patients with unilateral THR implants were evaluated over the period 1988–2008 (n = 20,814) (Table 2). The reason for this is that the side of the operation (left/right) is not reliably coded in the Hospital Discharge Register. If the data for the side of the operation are unavailable, then it is not possible to compare left-side with right-side THR from the registry data with any certainty.

**Table 2.** Number (N_a_) and percentage of replacements in cohorts when analyzing length of stay and unscheduled re-admissions, and number (N_b_) and percentage of replacements in cohorts when analyzing dislocations, re-operations, and infections

Cohort	No. (N_a_) of hips (%)	No. (N_b_) of hips (%)
1998	2,863 (10)	1,906 (9)
1999	3,083 (10)	2,025 (10)
2000	3,360 (11)	2,178 (11)
2001	3,554 (12)	2,389 (12)
2002	3,877 (13)	2,639 (13)
2003	4,336 (14)	3,056 (15)
2004	4,113 (14)	2,963 (14)
2005	5,080 (17)	3,658 (18)
Total	30,266 (100)	20,814 (100)

### Exclusion criteria

THRs for secondary OA, reoperations, and revisions of an implant were excluded (Table 3, see supplementary data). The manifestation of the diagnosis of secondary hip OA was noted retrospectively from the beginning of 1988. A patient was excluded from the study if there was a diagnosis of secondary hip OA in the Hospital Discharge Register between the beginning of 1988 and the day of the operation. Patients who were eligible for reimbursement in the Social Insurance Institution database for the sequelae of transplantation, uremia requiring dialysis, rheumatoid arthritis, or connective tissue disease were also excluded from the study. Patients with a simultaneously performed hip and knee arthroplasty were also excluded.

### Dislocations

We defined a reduction of a dislocated THR in 2 ways. Firstly, a reduction of a dislocated THR was considered to have been performed if there was a notification in the Hospital Discharge Register that either an open or closed reduction of a dislocated total hip prosthesis had been performed (NFH30 or NFH32), associated with a diagnosis of an internal mechanical complication of endoprosthesis (ICD10: T84.0). Secondly, closed reduction may also have been performed in the emergency room under light sedation anesthesia. These patients are often discharged from the accident and emergency units after closed reduction without an overnight stay in the hospital. According to Finnish regulations, it is mandatory to compile statistics on diagnosis codes in the accident and emergency units but relevant operational codes are not recorded routinely. Thus, a closed reduction of a dislocated THR was considered to have been performed if the patient had an unscheduled re-admission with a diagnosis code of mechanical complication of endoprosthesis (ICD: T84.0) but without any actual hospital admission. We believe that most of these non-admission cases are true dislocations, not periprosthetic fractures or aseptic loosening of the implant.

### Infections

A diagnosis code for deep prosthetic infection (ICD-10: T84.5) was used in the data search from the beginning of the surgical treatment period to the end of the follow-up. Data from the Finnish Arthroplasty Register were used to determine reoperations performed due to deep prosthetic infections.

### Unscheduled re-admission

An unscheduled re-admission was recorded if a patient was re-admitted to hospital or had required medical attention in an outpatient department or an accident and emergency unit of any hospital in Finland. Such an unscheduled re-admission also had to occur within the first 14 and the first 42 days from the end of the surgical treatment.

### Costs

The total cost of 1 day of treatment in a specialized care facility was used to calculate the potential savings if all THRs had been performed in the group of hospitals with the shortest LOS. The price of 1 day of treatment in hospitals performing THRs was on average 527 euros in Helsinki and Uusimaa district (HUS) during the period 2003–2005 (Peltola 2008). However, the mean cost of 1 day of treatment in hospitals performing THRs was not available for the whole country. The price of 1 re-admission in a specialized care facility was used to calculate increase in the costs if all THRs had been performed in the group of hospitals with the highest amount of re-admissions at 2 weeks. In 2006, the price of 1 re-admission in a specialized care facility was 278 euros on average in the whole country (Hujanen et al. 2008).

### Statistics

A logistic regression model for data at the individual level was used for dichotomous dependent variables and a generalized linear model (gamma-distribution, log-link) was used for continuous dependent variables, with hospital volume (classified) as explanatory variable. To improve the comparability of the study data, the analyses were adjusted for confounding factors (Iezzoni 2003). In addition, 95% confidence intervals (CIs) were determined. The age of the patient (under 40 years, over 40 years (divided into 9 incremental groups each of 5-year intervals up to 85 years), and over 85 years), sex, any previous THR, and co-morbidities were also adjusted for. In the models of LOS and LUIC, operation year dummies were included. We also performed calculations using head size of the prostheses as an adjustment factor to eliminate the effect of head size on dislocation rate. Co-morbidities were determined using the diagnoses obtained from the Hospital Discharge Register from the beginning of 1987 to the date of operation. In addition, the Social Insurance Institution database for eligibility for reimbursement including the use and cost of drugs was used to adjust for co-morbidity (Table 4). The illnesses chosen for adjustment were such that they might have had an effect on the performance of THR, on length of stay in the hospital, or on the rate of complications. The length of follow-up was also identified as a confounding factor for adjustment of the rates of complications.

**Table 4.** Related diseases used in the adjustment of the study population

*Hypertension* (ICD-10: I10*-I15*, ICD-9: 40*, Social Insurance Institutions entitlement to reimbursement: 205, use and cost of drugs ATC: C03*, C07* (if there is no coronary disease or atrial fibrillation), C09A*, C09B*, C09C*, C09D*, C08*)
*Coronary disease* (ICD-10: I20*-I25*, ICD-9: 410*-414*, Social Insurance Institutions entitlement to reimbursement: 206, 213, 280)
*Atrial fibrillation* (ICD-10: I48*, ICD-9: 4273*, Social Insurance Institutions entitlement to reimbursement: 207, use and cost of drugs ATC: B01AA03)
*Heart insufficiency* (ICD-10: I50*, ICD-9: 428*, Social Insurance Institutions entitlement to reimbursement: 201)
*Diabetes* (ICD-10: E10*-E14*, ICD-9: 250*, Social Insurance Institutions entitlement to reimbursement: 103 use and cost of drugs ATC-DDD: A10A*, A10B*)
*Cancer* (ICD-10: C00*-C99*, D00*-D09*, ICD-9: 140*-208*, Social Insurance Institutions entitlement to reimbursement: 115, 116, 117, 128, 130, 180, 184, 185, 189, 311, 312, 316, use and cost of drugs ATC L01* except L01BA01)
*COPD and asthma* (ICD-10: J44*-J46*, ICD-9: 4912*, 496*, 493*, Social Insurance Institutions entitlement to reimbursement: 203, use and cost of drugs ATC: R03*)
*Depression* (ICD-10: F32*-F34*, ICD-9: 2960*, 2961*, 2069*, use and cost of drugs ATC: N06A*)
*Parkinson's disease* (ICD-10: G20*, ICD-9: 332*, Social Insurance Institutions entitlement to reimbursement: 110, use and cost of drugs ATC: N04B*)
*Dementia* (ICD-10: F00*-F03*, G30*, ICD-9: 290*, 3310*, Social Insurance Institutions entitlement to reimbursement: 307, use and cost of drugs ATC: N06D*)
*Kidney insufficiency* (ICD-10: N18*, ICD-9: 585*, Social Insurance Institutions entitlement to reimbursement: 137)
*Mental disorders* (ICD-10: F20*-F31*, ICD-9: 295*-298*, except 2960*, 2961*, Social Insurance Institutions entitlement to reimbursement: N05A* except N05AB04 and N05AB01 and there is no dementia)

## Results

### Length of stay (LOS) and length of uninterrupted institutional care (LUIC) (Table 5)

In 2005, LOS was 5.49 days in group 4, 6.65 days in group 3, 7.63 days in group 2, and 6.84 days in group 1 (the reference group). During the whole study period, LOS was longer in group 1 than in group 4 (p < 0.001). However, LOS was shorter in group 1 than in group 2 (p < 0.001). In 2005, LUIC was 9.91 days in group 4, 10.47 days in group 3, 10.59 days in group 2, and 10.27 days in group 1. During the whole study period, LUIC was shorter in group 4 than in group 1 (p < 0.001). Nonetheless, LUIC was longer in group 3 and in group 2 than in group 1 (p < 0.001 for both comparisons).

**Table 5.** The average length of stay and the number of days saved if patients were treated in hospitals with the shortest length of stay

Year	Group	LOS	N	Difference in LOS	Days saved
1998	1	10.06	233	0.35	82
	2	10.52	1,395	0.81	1,130
	3	9.05	1,145	-0.66	-756
	4	9.71	90	–	–
	In total		2,863		456
1999	1	9.80	227	0.59	134
	2	10.01	1,664	0.8	1,331
	3	8.85	953	-0.36	-343
	4	9.21	239	–	–
	In total		3,083		1,122
2000	1	9.37	388	1.50	582
	2	9.81	1,452	1.94	2,817
	3	8.82	932	0.95	885
	4	7.87	588	–	–
	In total		3,360		4,284
2001	1	8.65	493	0.92	454
	2	9.66	1,386	1.93	2,675
	3	8.57	899	0.84	755
	4	7.73	776	–	–
	In total		3,554		3,884
2002	1	8.34	449	1.21	543
	2	9.15	1,647	2.02	3,327
	3	7.76	1,207	0.63	760
	4	7.13	574	–	–
	In total		3,877		4,630
2003	1	7.17	315	1.08	340
	2	8.58	1,358	2.49	3,381
	3	7.93	988	1.84	1,818
	4	6.09	1,675	–	–
	In total		4,336		5,539
2004	1	7.22	346	1.34	464
	2	8.46	1,181	2.58	3047
	3	7.38	1,059	1.50	1,589
	4	5.88	1,527	–	–
	In total		4,113		5,100
2005	1	6.84	308	1.35	416
	2	7.63	1,508	2.14	3,227
	3	6.65	951	1.16	1,103
	4	5.49	2,313	–	–
	In total		5,080		4,746
1998–2005					29,761

LOS: annual mean length of stay (surgical treatment period) in days; N: number of patients; Difference in LOS: difference between the shortest length of stay and that of other hospital groups in days; Days saved: number of days saved if patients were operated in hospitals with the shortest length of stay (days saved = difference in LOS multiplied by N). In 1998 and 1999, the average LOS was shorter in group 3 than in group 4. This is why “Difference in LOS” and “Days saved” have negative values in the Table.

Theoretically, if all THRs during the study period in Finland had been performed in the very–high-volume hospitals with the shortest length of stay, total LOS in the hospital would have decreased by 29,761 days (1.0 day per patient). Thus, the costs would have been reduced by 15,684,047 euros.

If all THRs during the study period in Finland had been performed in the very–high-volume hospitals, this would have led to a total of 355 new re-admissions at 2 weeks. The increase in cost for these re-admissions would have been 98,690 euros.

If we then subtract the increased costs due to a higher rate of re-admissions (98,690 euros) from the total savings in LOS (15,684,047 euros), the final savings during the follow-up time would have been 15,585,357 euros.

### Complications (Table 6)

In unadjusted data, statistically there were significantly less dislocations in groups 3 and 4 than in group 1. There were also less reoperations in group 4 than in group 1. In group 1, there were more re-admissions both at 2 and 6 weeks than in group 1. However, in adjusted data there was only a trend of fewer re-admissions within 14 days in group 1 than in group 4. There were significantly more dislocations in group 1 than in group 3. There was no association between hospital volume and reoperation rates or infection rates when using adjusted data.

**Table 6a.** Unadjusted odds ratios for unscheduled re-admissions within 14 and 42 days, and for dislocations, reoperations, and infections

	Re-admsissions 14 days		Re-admsissions 42 days		Dislocations		Reoperations		Infections	
Groups	OR	95% WCI	OR	95% WCI	OR	95% WCI	OR	95% WCI	OR	95% WCI
2 vs. 1	0.9	0.8–1.1	0.9	0.8–1.0	0.9	0.7–1.2	1.1	0.9–1.3	1.0	0.7–1.6
3 vs. 1	1.0	0.9–1.2	1.0	0.8–1.1	0.7	0.6–0.9	1.0	0.8–1.2	0.9	0.6–1.5
4 vs. 1	1.2	1.0–1.4	1.1	1.0–1.3	0.8	0.6–1.0	0.7	0.5–0.9	0.8	0.5–1.3

OR: odds ratio; 95% WCI: 95% Wald confidence interval. See Methods for explanation of groups.

**Table 6b.** Adjusted odds ratios for unscheduled re-admissions within 14 and 42 days, and for dislocations, reoperations, and infections

	Re-admsissions 14 days		Re-admsissions 42 days		Dislocations		Reoperations		Infections	
Groups	OR	95% WCI	OR	95% WCI	OR	95% WCI	OR	95% WCI	OR	95% WCI
2 vs. 1	0.9	0.8–1.1	0.9	0.8–1.0	0.9	0.7–1.1	1.0	0.8–1.3	1.0	0.6–1.6
3 vs. 1	1.0	0.9–1.2	0.9	0.8–1.1	0.7	0.6–0.9	0.9	0.8–1.2	0.9	0.6–1.4
4 vs. 1	1.2	1.0–1.4	1.1	1.0–1.2	1.1	0.9–1.4	0.9	0.7–1.1	0.8	0.5–1.4

OR: odds ratio; 95% WCI: 95% Wald confidence interval. See Methods for explanation of groups.

We also performed the analyses using the ownership of the hospitals (private or public) as a confounding factor. Of all 30, 266 hips studied, 4,150 hips were operated on in private hospitals. There was no significant difference between hospital volume results when the private/public dichotomy was included in the model. LOS was statistically significantly shorter in private hospitals than in public hospitals.

## Discussion

Our findings indicate that specialization of hip replacements in high-volume hospitals should reduce costs by shortening LOS, and may reduce the dislocation rate.

### Validity of the data

The reporting accuracy of the Hospital Discharge Register in Finland is high when considering surgical operations. In the late 1980s, at least 95% of operations were already being recorded in this register (Keskimäki and Aro 1991). The correlation between the Finnish Discharge Register and the Finnish Arthroplasty Register is high (Peltola 2008). The strength of our study is that it included operative data from both private and public hospitals. We adjusted for patient age, sex, surgery, and medical diagnosis. Adjustment calculations were also done for the head size of the prosthesis. However, not all factors associated with dislocation rate, such as surgical approach, could be adjusted for in the data from the current study.

Our basic assumption was that performing more hip surgery correlates with skills. Change of hospital volume group would not interfere with our analyses if hospital volume is an important factor compared to other variables affecting the quality of hip surgery.

### Length of stay

The LOS after THR varies considerably. There are reports in the literature that THRs have actually been performed as day-case surgery (Berger et al. 2004), even though very early discharge has been cautioned against (Parvizi et al. 2007). Nevertheless, reducing LOS reduces the cost of care and permits increase in bed occupancy rates (Williams et al. 2005). In previous studies, longer LOSs after THR have been associated with lower provider volumes (Lavernia and Guzman 1995, Doro et al. 2006, Judge et. al. 2006). In our study, not only was a very high hospital volume associated with shorter surgical treatment periods but also with shorter LUIC. Large amounts of money can be saved if THRs are performed in very–high-volume hospitals. However, when the postoperative care is made more efficient, the easiest and cheapest days are removed, not the heaviest days including the first postoperative day and the discharge day. In the near future in Finland, a large number of people born in the late 1940s and early 1950s will retire. Because of the aging population and the decreasing number of nurses, more patients will have to be treated using the same resources as those used today—by optimizing efficiency.

### Unscheduled re-admissions

Unscheduled re-admission rate is a national key performance indicator used by the UK Department of Health (Adeyemo and Radley 2007). A 28-day emergency re-admission rate has been used as a clinical indicator to compare surgical and orthopedic performance between trusts in England and in Scotland (Courtney et al. 2003). In a study by Cullen et al. (2006), 9% of patients were re-admitted within 28 days of discharge after THR. The main reasons for re-admission were thromboembolism, dislocations, and wound complications. Reducing the length of stay lowered the cost of care per patient and permitted increased bed occupancy, but the effect on emergency re-admission rates was equivocal (Williams et al. 2005). The odds ratio of emergency re-admission for primary hip replacement was 0.54 when the length of stay was 4–7 days and 0.55 when it was 8–14 days, considering the odds ratio 1.0, when the length of stay was 4 days or under (Williams et al. 2005). In a study by Judge et al. (2006), there was evidence of a higher re-admission rate for high-volume trusts. In our study, the rate of re-admissions in the low-volume hospitals was also lower than in all other hospital groups. When the length of stay is longer, early problems manifest in the hospital and are treated immediately in situ; thus, re-admissions are less likely to occur than in shorter-stay facilities. However, the reasons for re-admissions are most often not major surgical complications but minor wound problems, suspicion of venous thromboembolism, and medication problems. The costs of re-admissions were low compared to the costs of longer length of stay.

### Dislocations

The dislocation rate during the first year after THR has been reported to range from less than 1% to almost 4% (Phillips et al. 2003, Khatod et al. 2006, Meek et al. 2006). Several factors are constantly reported to be statistically associated with THR dislocation rate. These include surgical diagnosis, femoral head size, patient age and sex, American Association of Anesthesiologists (ASA) score, cognitive dysfunction, surgical approach, surgeon volume, and hospital volume (Meek et al. 2006). It is not possible to influence all of these factors to reduce the number of dislocations. However, healthcare workers should try to manipulate those factors that can be influenced, such as surgeon volume, hospital volume, head size, and repair of soft tissues.

In our study, the low-volume hospitals had higher dislocation rates than the high-volume hospitals and the very–high-volume hospitals. However, the dislocation rate for very-high-volume hospitals was not lower than that of low-volume hospitals when we analyzed adjusted data. The patients in large-volume units are probably somewhat younger than in low-volume hospitals. In small units, there are more typical osteoarthritic patients of advanced age who are at risk of having short-term complications. On the other hand, very–high-volume hospitals are mainly university hospitals with junior surgeons who perform replacements as part of their education. Furthermore, not all diseases—for example, obesity and alcoholism—nor the condition of bone and soft tissues can be adjusted, and it is likely that even after adjustment, there will be more difficult patients in university hospitals that have adverse influential effects on the dislocation rates observed. High dislocation rates in low-volume hospitals are an alarming finding that has also been reported previously (Shervin et al. 2007).

### Reoperations

Instability has been the most common reason for early reoperations, whereas osteolysis and loosening are the most common reasons for late revisions (Clohisy et al. 2004). Because the follow-up time in our study was short, we assumed that many reoperations were performed either because of infections, instability, or periprosthetic fractures. Early dislocations after THR are most often treated by closed reduction; these were analyzed separately. Open reduction and revision operations as a treatment for dislocated hip prostheses are uncommon. Unfortunately, the reason for revision is not directly available from the Finnish Arthroplasty Register, only the type of revision performed (for example exchange of the stem with the diagnosis code NFC20, but not if the revision was performed for periprosthetic fracture or for dislocation, both of which have the diagnosis code T84.0). We found less reoperations in the very-high-volume group than in the low-volume group when using unadjusted raw data. However, this difference did not reach statistical significance when using the adjusted data. In a study by Manley et al. (2008), the patients of low-volume surgeons had a greater risk of arthroplasty revision at 6 months but no greater risk of revision at the time of longer-term follow-up. No associations between hospital volumes and the rates of revision of THA were found by Judge et al. (2006) and Manley et al. (2008).

### Infections

In recent studies, the rate of deep infection after THA has varied between 0.6% and 0.9% (Gastmeier et al. 2005, Muilwijk et al. 2006, Phillips et al. 2006). Independent risk factors for surgical site infections after THR are patient age, surgical diagnosis, ASA score, and duration of operation (Ridgeway et al. 2005). In a systematic literature review, no association between hospital volumes and infection rates was found (Shervin et al. 2007). Nowadays, deep infections after THR are rare that the capability of population-based studies to determine significant differences between hospitals is limited. In our study, patient age, sex, surgical diagnosis, and medical diagnoses were adjusted for. Thus, it was not surprising that no association between infection rate and hospital volume was detected.

### Summary

We found that LOS was shortest in the very-high-volume hospitals. The costs of more frequent re-admissions in the very–high-volume hospitals were low compared to the savings from short LOS. Specialization of high-volume hospitals regarding hip replacements should reduce costs by significantly shortening length of stay, and it may reduce the dislocation rate.

## Supplementary data

Table 3 is available at our website (www.actaorthop.org), identification number 3967.
