# Supplementary material for: The effect of hospital volume on length of stay, re-admissions, and complications of total hip arthroplasty: A population-based register analysis of 72 hospitals and 30,266 replacements
Source: Acta Orthop. 2011 Feb 10;82(1):20–6. doi: 10.3109/17453674.2010.533930 (PMC3229993; doi:10.3109/17453674.2010.533930)
Supplement: Supplementary file 1 [file ORT-1745-3674-82-020-s3967.pdf]

Supplementary article data

The effect of hospital volume on length of stay, re-admissions, and complications of total hip arthroplasty

A population-based register analysis of 72 hospitals and 30,266 replacements

Keijo T Mäkelä[1], Unto Häkkinen[2], Mikko Peltola[2], Miika Linna[2], Heikki Kröger[3], and Ville Remes[4]

[1]Department of Orthopaedics and Traumatology, Turku University Central Hospital; [2]National Institute for Health and Welfare; [3]Department of Orthopaedics and Traumatology, Kuopio University Hospital[3]; [4]Department of Orthopaedics and Traumatology, Peijas Hospital, Helsinki University Central Hospital, Finland
Correspondence: keijo.makela@tyks.fi
Submitted 10-02-08. Accepted 10-07-17

Table 3. Exclusion criteria (ICD-10 diagnosis)

S72.0	Fracture of neck of femur
S72.1	Pertrochanteric fracture of femur
S72.2	Subtrochanteric fracture of femur
M91.1	Juvenile osteochondrosis of head of femur (Legg-Calvé-Perthes)
M93.0	Slipped upper femoral epiphysis (nontraumatic)
S324	Fracture of acetabulum
M45.*	Ankylosing spondylitis
Q65.*	Luxatio coxae congenita
M16.4	Post-traumatic coxarthrosis, bilateral
M16.5	Other post-traumatic coxarthrosis
M16.6	Other secondary coxarthrosis, bilateral
M16.7	Other secondary coxarthrosis
M16.9	Coxarthrosis, unspecified
M87.*	Osteonecrosis
M00.*	Pyogenic arthritis
M05.*	Seropositive rheumatoid arthritis
M06.*	Other rheumatoid arthritis
M07.*	Psoriatic and enteropathic arthropathies
M08.*	Juvenile arthritis
D66.	Hereditary factor VIII deficiency
D67.	Hereditary factor IX deficiency
D68.	Other coagulation defects
M36.2	Haemophilic arthropathy
Q77.	Osteochondrodysplasia with defects of growth of tubular bones and spine
Q78.	Other osteochondrodysplasias
Q79.	Congenital malformations of the musculoskeletal system, not elsewhere classified

Open Access - This article is distributed under the terms of the Creative Commons Attribution Noncommercial License which permits any noncommercial use, distribution, and reproduction in any medium, provided the source is credited.
DOI 10.3109/17453674.2010.533930
